# Wide‐Field Imaging of Retinal Vascular Macroaneurysm Associated With Retinal Racemose Hemangioma

**DOI:** 10.1155/crop/1972222

**Published:** 2025-12-06

**Authors:** Yamato Katsura, Hisashi Fukuyama, Fumi Gomi

**Affiliations:** ^1^ Department of Ophthalmology, Hyogo Medical University, Nishinomiya, Japan, hyo-med.ac.jp

## Abstract

**Purpose:**

The purpose of this study is to describe a case of exudative retinal vascular macroaneurysm (RVM) associated with retinal racemose hemangioma (RRH).

**Methods:**

The study design is an observational case report.

**Results:**

A 40‐year‐old woman presented with visual field defects in her right eye. Fundus examination revealed exudative RVM in combination with RRH. Subsequently, subretinal hemorrhage extended toward the macula, and her best‐corrected visual acuity declined to 20/63. Treatment consisted of intravitreal bevacizumab injections and retinal photocoagulation for the RVM. This approach led to resolution of the RVM, confirmed by OCTA. Eight months after photocoagulation, her best‐corrected visual acuity improved to 20/20.

**Conclusion:**

This case demonstrates the effectiveness of combined intravitreal bevacizumab injections and retinal photocoagulation for treating RVM. The resolution of the RVM was confirmed by OCTA. Wide‐field OCTA is indispensable for evaluating the extent of arteriovenous malformations and for tracking post‐treatment changes and complications. Considering the potential for recurrence of RVM associated with RRH, careful and ongoing follow‐up is necessary.

## 1. Introduction

Retinal racemose hemangioma (RRH) is a rare congenital and nonhereditary type of retinal arteriovenous malformation (RAVM) characterized by direct anastomosis between retinal arteries and veins [1]. Patients with RRH often exhibit associated brain abnormalities, a condition known as Wyburn–Mason syndrome. Although RRH frequently remains asymptomatic, some patients develop retinal complications such as retinal vein occlusion, vitreous hemorrhage, or retinal vascular macroaneurysm (RVM). This report describes a case of RVM associated with RRH, which was treated using a combination of intravitreal bevacizumab injections and retinal photocoagulation therapy. Treatment efficacy was assessed with wide‐field fundus imaging and optical coherence tomography angiography (OCTA).

## 2. Case Report

A 40‐year‐old woman was referred to Hyogo Medical University Hospital with complaints of visual field loss in her right eye. Best‐corrected visual acuity in the right eye was 20/16. Fundus examination revealed enlarged and tortuous vessels originating from RRH, along with RVM and subretinal hemorrhage located superior to the macular region (Figure 1a). Vertical optical coherence tomography (OCT) imaging highlighted dilated vessels corresponding to RRH and the subretinal hemorrhage (Figure 1b). Examination of the left eye showed no abnormalities.

Figure 1(a) Ultrawide‐field fundus photograph (Optos California, Nikon) showing dilated and tortuous arteriovenous malformations caused by retinal racemose hemangioma, along with a hemorrhagic retinal artery macroaneurysm. (b) Vertical optical coherence tomography displaying dilated vessels.

**Figure:**
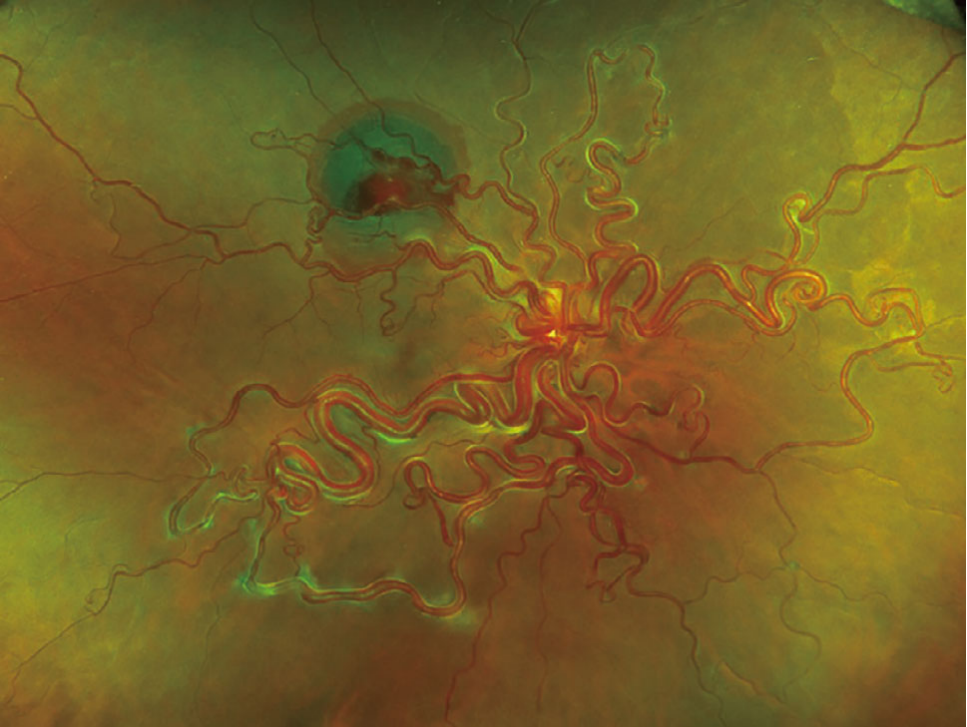
(a)

**Figure:**
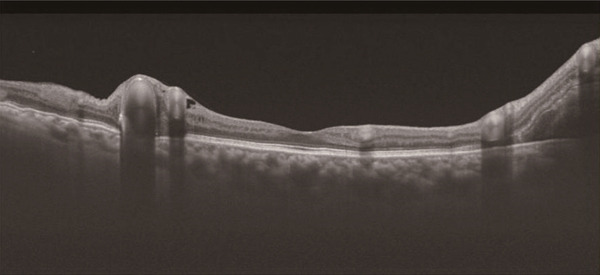
(b)

A detailed medical history revealed no personal or family history of related conditions; prior brain magnetic resonance imaging revealed no abnormalities. Fluorescein angiography demonstrated early filling of dilated retinal vessels before choroidal filling (choroidal flush) and direct continuation between retinal arteries and veins (Figure 2a). Twenty seconds later, RVM and the entire retinal vasculature became clearly visible (Figure 2b).

Figure 2Ultrawide‐field fluorescein angiography: (a) At 7 s after dye injection, the image shows the direct connection between abnormally dilated retinal arteries and veins, appearing before choroidal fluorescence. (b) At 27 s, the retinal arterial macroaneurysm is fully outlined (red circle).

**Figure:**
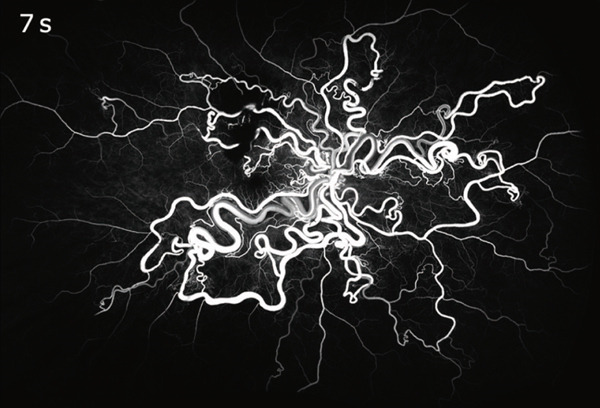
(a)

**Figure:**
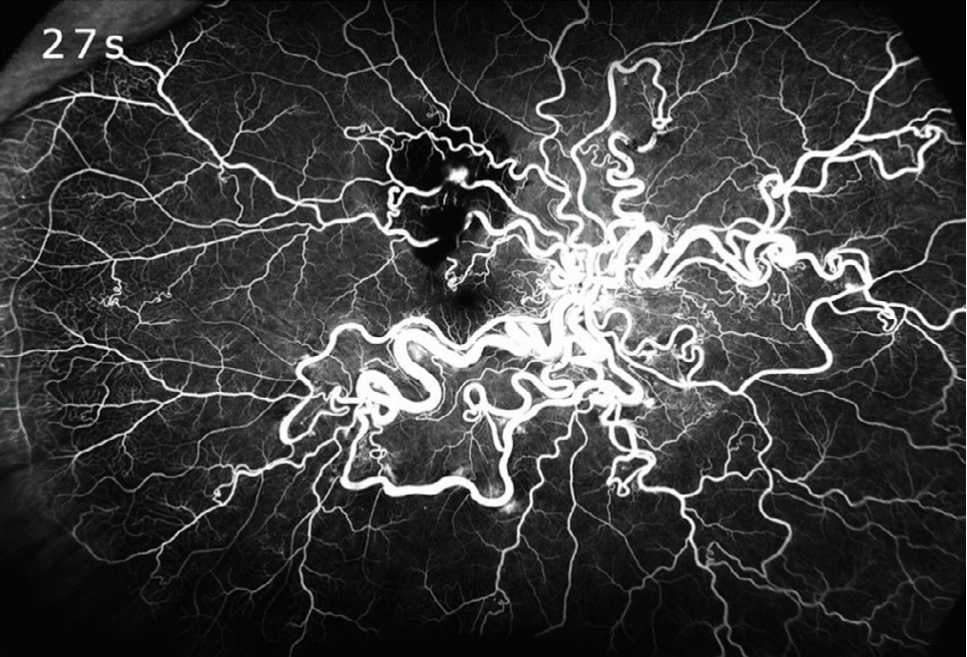
(b)

**Figure 4:**
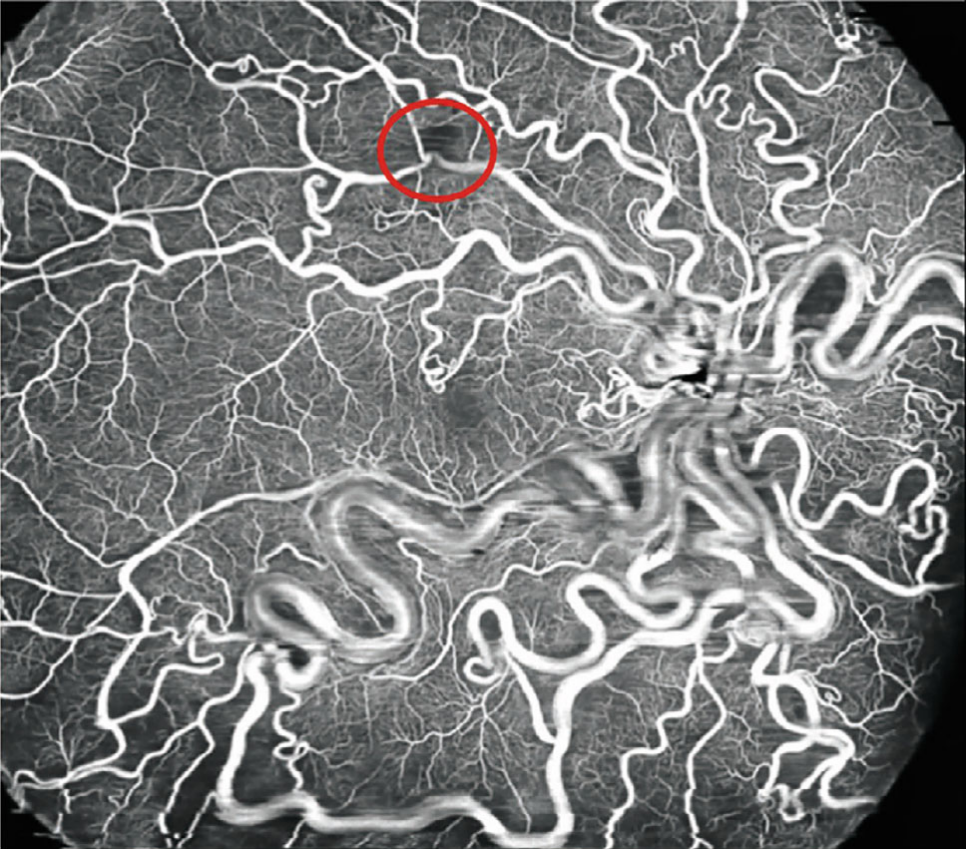
A 20 × 23 mm optical coherence tomography angiography image (Xephilio OCT‐S1, Canon) after combined therapy involving laser photocoagulation and bevacizumab. The image shows a kinked vessel without any signals corresponding to the retinal arterial macroaneurysm (red circle). It clearly shows abnormally dilated vessels with the arteriovenous malformation in a wide‐field view.

One month after the patient’s initial visit, subretinal hemorrhage and exudation from the RVM had expanded, prompting an intravitreal bevacizumab injection (1.25 mg/in 0.05 mL). At that time, visual acuity remained 20/16. Two weeks later, the patient’s visual acuity declined to 20/63 as the subretinal hemorrhage extended to involve the subfoveal region (Figure 3a,b). Retinal photocoagulation targeting the RVM was subsequently performed in conjunction with a second bevacizumab injection. One month after photocoagulation, the subretinal hemorrhage and exudation had diminished. By 3 months after photocoagulation, fundus examination showed resolution of the subretinal hemorrhage with whitening of the RVM vessels, and OCT confirmed the disappearance of subfoveal exudation (Figure 3c,d). At this point, wide‐field OCTA revealed the absence of blood flow within the RVM (Figure 4). Eight months after photocoagulation, the patient’s best‐corrected visual acuity had improved to 20/20. During 18 months of follow‐up, no RVM recurrence was observed.

Figure 3Time course of fundus (a, c) and OCT (b, d) images. (a, b) Six weeks after the initial visit, subretinal hemorrhage continued to expand, involving the macula despite the first intravitreal injection of bevacizumab. Retinal photocoagulation was subsequently performed for the retinal vascular macroaneurysm (RVM). (c, d) Three months after combined therapy involving laser photocoagulation and bevacizumab, the RVM became less prominent; whitening of the peripheral vessels was evident. Subretinal hemorrhage and exudation showed resolution.

**Figure:**
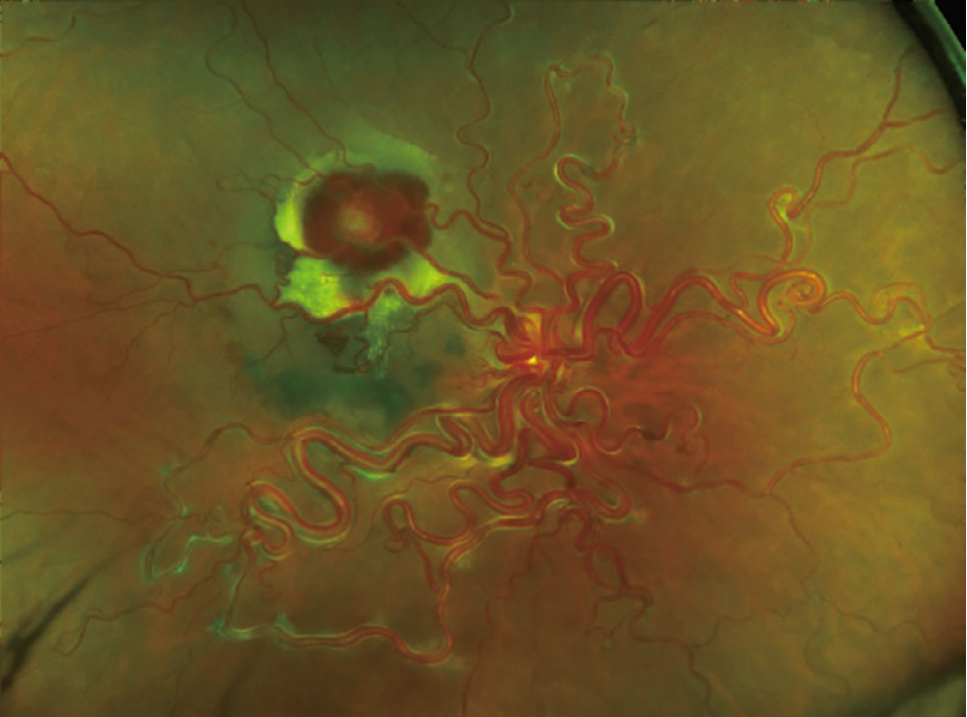
(a)

**Figure:**
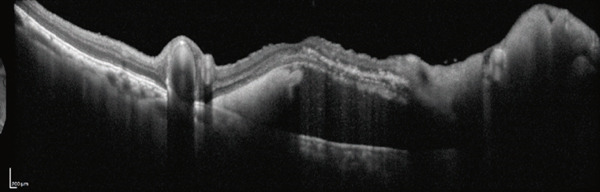
(b)

**Figure:**
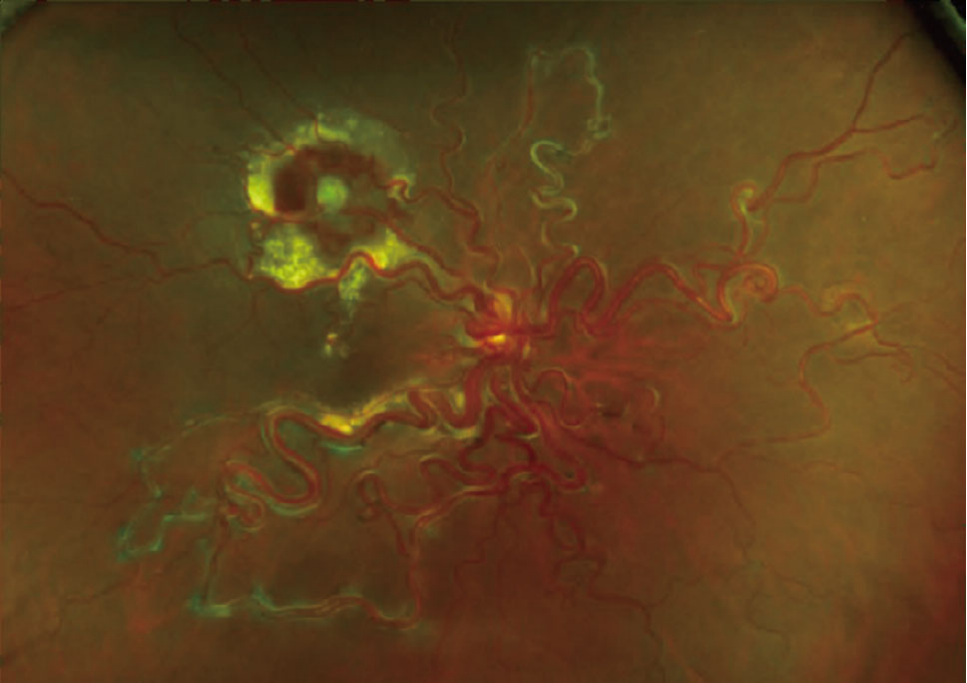
(c)

**Figure:**
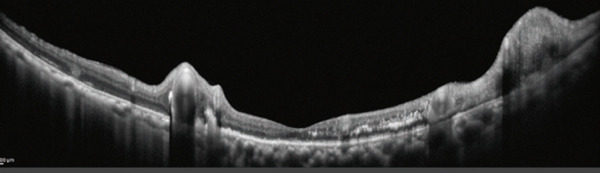
(d)

## 3. Discussion

RRH is a congenital arteriovenous malformation characterized by direct anastomosis between retinal arterioles and venules. It is classified into three types based on the size and location of the vascular malformation. In the present case, extensive arteriovenous malformations with indistinct, dilated, and tortuous vessels were observed, consistent with Type 3 RRH. This type carries increased risk of retinal complications, such as retinal vein occlusion, macular edema, and vitreous hemorrhage [2]. Although this case involved a ruptured RVM associated with RRH, RVM is not a common complication of RRH. In a study by Qin et al. [3], only one case (0.6%) of RVM was reported among 167 RRH cases with complications. However, several reports have described exudative or hemorrhagic RVM in eyes with RRH. RVM formation may be related to hyperdynamic blood flow arising from the “steal phenomenon,” which involves increased venous pressure and reduced arterial pressure within tortuous vessels [4–7]. These conditions may lead to focal turbulence in blood flow, contributing to RVM formation.

To address the hemorrhagic RVM, we initially administered an intravitreal injection of bevacizumab to promote the resolution of hemorrhage and exudation from RVM as previously reported [5, 8]. Because the antivascular endothelial growth factor (VEGF) monotherapy was not sufficient to resolve RVM, photocoagulation to RVM was promptly conducted to anticipate earlier visual recovery combined with the second injection of bevacizumab. Here, bevacizumab was used to reduce further bleeding from the RVM damaged by photocoagulation and suppress inflammatory activity. This approach led to resolution of the subretinal hemorrhage and fluid, as well as scarring of the RVM.

OCTA provides a noninvasive method for visualizing retinal vessels by detecting blood flow [9]. In this report, wide‐field OCTA successfully visualized the entire vascular malformations characteristic and retinal capillary abnormalities of RRH although dye angiography was essential to detect abnormal blood circulation within RRH. We previously showed that OCTA can confirm the success of direct photocoagulation of leaking retinal microaneurysms by demonstrating the absence of blood flow within treated areas [10]. In the present case, OCTA helped to confirm the resolution of RVM after treatment. Another study also revealed time‐dependent changes in RVM associated with RRH via 9 × 9 mm OCTA [6].

Because RVM associated with RRH can recur, regular monitoring of vascular abnormalities is essential. Considering that RRH can affect large portions of the retina, wide‐field imaging is invaluable for efforts to evaluate lesion extent, track post‐treatment changes, and identify potential complications noninvasively.

## Consent

No written consent has been obtained from the patients as there is no patient identifiable data included in this case report.

## Conflicts of Interest

The authors declare no conflicts of interest.

## Funding

No funding was received for this manuscript.

## Data Availability

All data supporting the findings of this study are included within the article.
